# Supplementary material for: Capillary Flow-Driven and Magnetically Actuated Multi-Use Wax Valves for Controlled Sealing and Releasing of Fluids on Centrifugal Microfluidic Platforms
Source: Micromachines (Basel). 2022 Feb 16;13(2):303. doi: 10.3390/mi13020303 (PMC8877048; doi:10.3390/mi13020303)
Supplement: Supplementary file 1 [file micromachines-13-00303-s001.zip › micromachines-1585547-supplementary.pptx]

## Slide 1
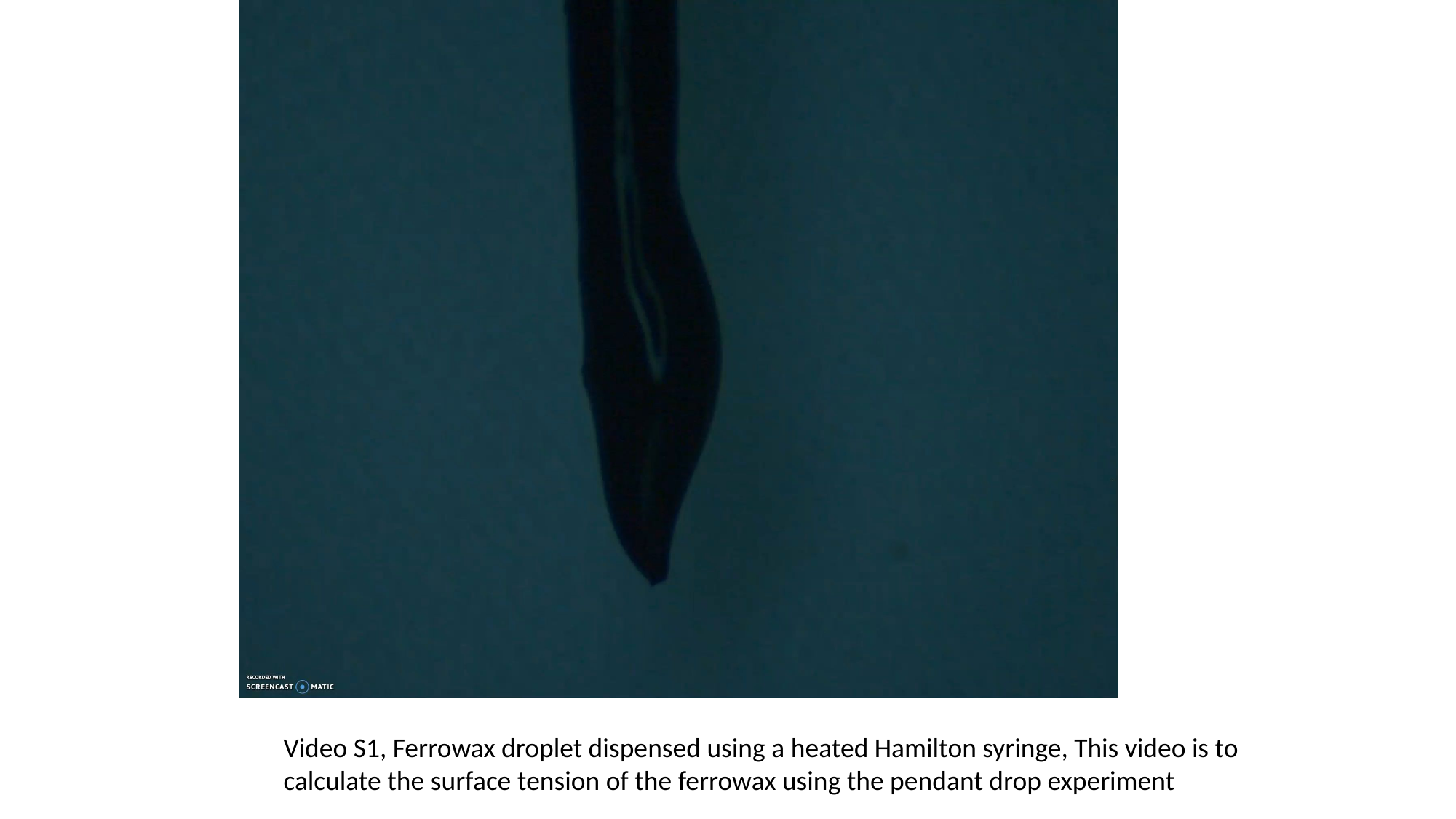

#
Video S1, Ferrowax droplet dispensed using a heated Hamilton syringe, This video is to calculate the surface tension of the ferrowax using the pendant drop experiment

## Slide 2
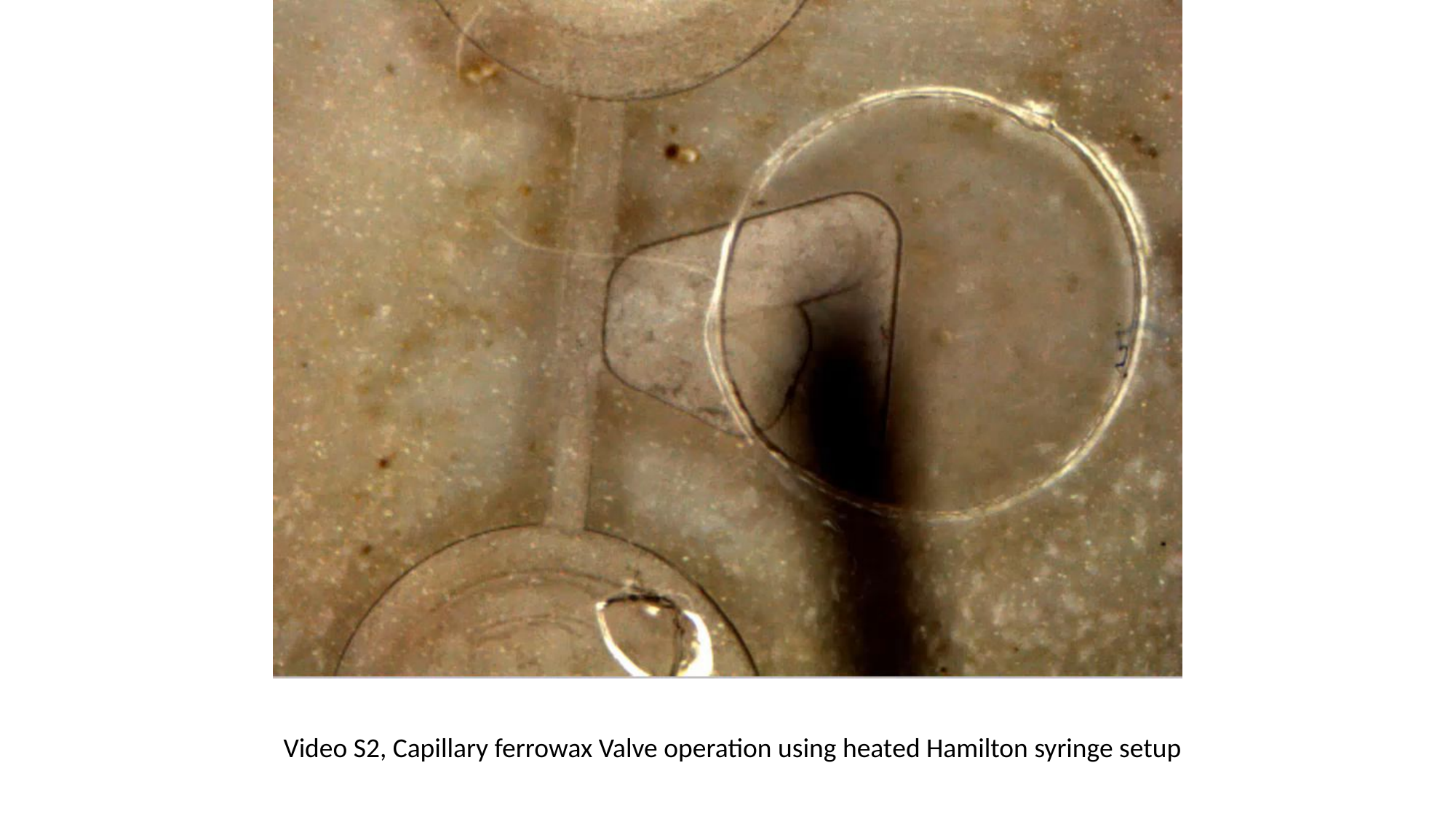

Video S2, Capillary ferrowax Valve operation using heated Hamilton syringe setup

## Slide 3
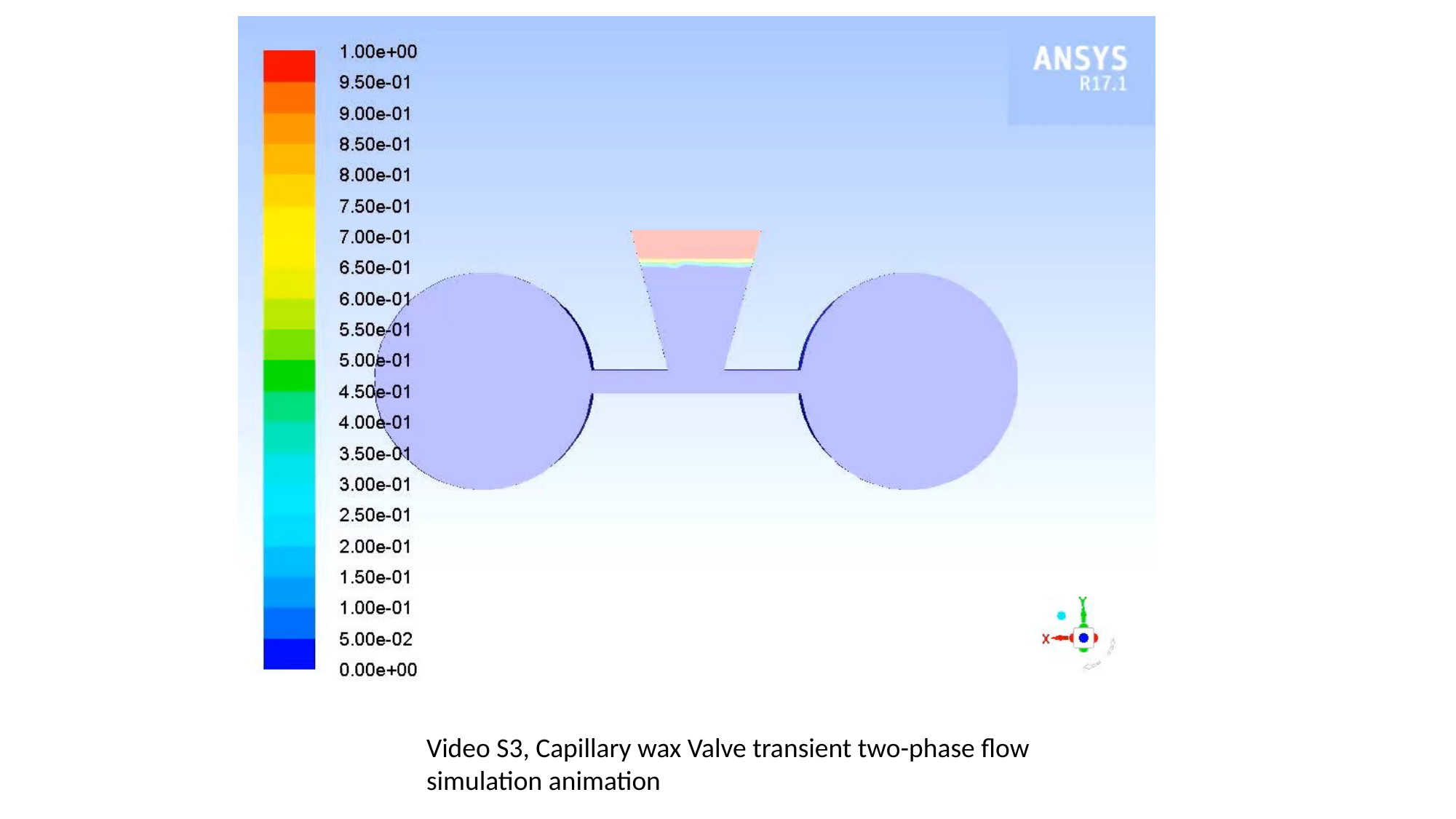

Video S3, Capillary wax Valve transient two-phase flow simulation animation

## Slide 4
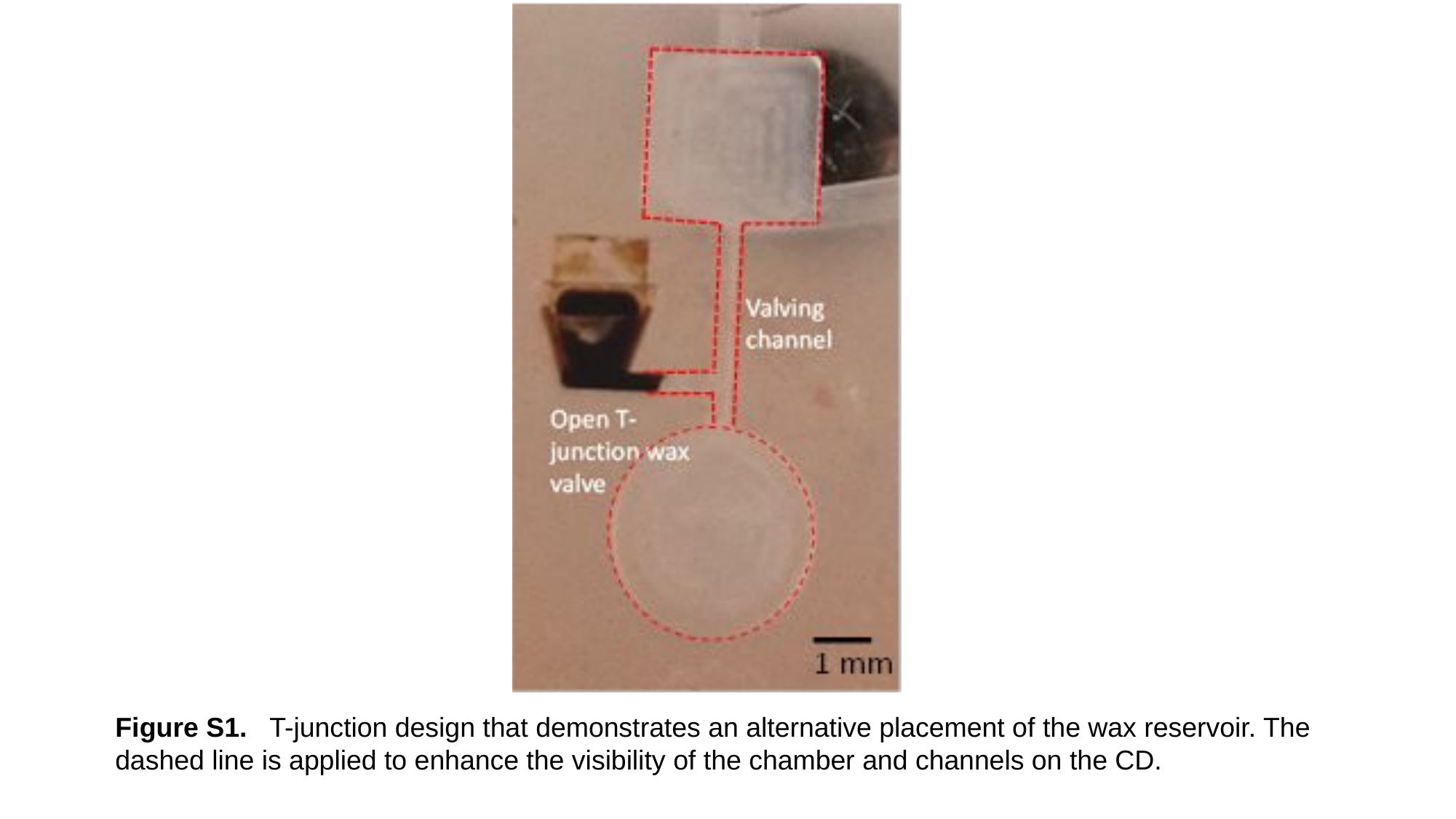

Figure S1. T-junction design that demonstrates an alternative placement of the wax reservoir. The dashed line is applied to enhance the visibility of the chamber and channels on the CD.
